# Cost and utilization analysis of concurrent versus staged testicular prosthesis implantation for radical orchiectomy

**DOI:** 10.1371/journal.pone.0296735

**Published:** 2024-01-08

**Authors:** Vi Nguyen, Arman Walia, Joshua J. Horns, Niraj Paudel, Aditya Bagrodia, Darshan P. Patel, Tung-Chin Hsieh, James M. Hotaling

**Affiliations:** 1 Department of Urology, University of California, San Diego, San Diego, California, United States of America; 2 Department of Urology, University of Utah, Salt Lake City, Utah, United States of America; Icahn School of Medicine at Mount Sinai Department of Pharmacological Sciences, UNITED STATES

## Abstract

**Purpose:**

American Urological Association guidelines recommend testicular prosthesis discussion prior to orchiectomy. Utilization may be low. We compared outcomes and care utilization between concurrent implant (CI) and staged implant (SI) insertion after radical orchiectomy.

**Materials & methods:**

The MarketScan Commercial claims database (2008–2017) was queried for men ages >18 years who underwent radical orchiectomy for testicular mass, stratified as orchiectomy with no implant, CI, or SI. 90-day outcomes included rate of reoperation, readmission, emergency department (ED) presentation, and outpatient visits. Regression models provided rate ratio comparison.

**Results:**

8803 patients (8564 no implant, 190 CI, 49 SI; 2.7% implant rate) were identified with no difference in age, Charlson Comorbidity Index, insurance plan, additional cancer treatment, or metastasis. Median perioperative cost at orchiectomy (+/- implant) for no implant, CI, and SI were $5682 (3648–8554), $7823 (5403–10973), and $5380 (4130–10521), respectively (p<0.001). Median perioperative cost for SI at implantation was $8180 (4920–14591) for a total cost (orchiectomy + implant) of $13650 (5380 + 8180). CI patients were more likely to have follow-up (p = 0.006) with more visits (p = 0.030) compared to the SI group post-implantation but had similar follow-up (p = 0.065) and less visits (p = 0.025) compared to the SI patients’ post-orchiectomy period. Overall explant rates were 4.7% for CI and 14.3% for SI (p = 0.04) with a median time to explant of 166 (IQR: 135–210) and 40 days (IQR: 9.5–141.5; p = 0.06). Median cost of removal was $2060 (IQR: 967–2880).

**Conclusions:**

CI placement has less total perioperative cost, lower explant rate, and similar postoperative utilization to SI.

## Introduction

Testicular cancer is the most common solid tumor malignancy among young males ages 20–39 [1]. The American Urological Association (AUA) Guidelines for Early Stage Testicular Cancer strongly recommend radical inguinal orchiectomy for patients with a testicular mass, and this malignancy is highly curable with a 10-year survival rate approaching 95% [2, 3]. Given the high incidence with prolonged survival in young men coupled with the loss of an organ associated with self-esteem, sexuality, and masculinity, it is critical to address the psychologic sequelae and quality of life among long-term survivors of testicular cancer [4–6]. This population has been shown to experience negative changes in body image associated with sexual dysfunction including reduced sexual interest, activity and enjoyment, erectile or ejaculatory dysfunction, and increased sexual discomfort [7].

Testicular prostheses can restore self-image and improve quality of life among patients who have undergone orchiectomy, and satisfaction rates are high, with 71–88% of patients reporting they would undergo implantation again and 79% reporting they would recommend implantation to others [8–10]. Despite the AUA guideline recommendation for discussion of testicular prostheses prior to orchiectomy for testicular cancer, approximately 50% of patients report they were not offered implantation before their surgery [2, 9, 11].

Preoperative counseling regarding testicular prostheses represents an area of clinical improvement, but it remains unknown if the surgical approach of a concurrent implant (CI) versus staged implant (SI) is more desirable. We sought to investigate differences in cost and care utilization between CI and SI insertion after radical orchiectomy. We hypothesized that CI insertion was less costly with non-inferior clinical outcomes, therefore further supporting its value in preoperative counseling.

## Materials and methods

### Cohort identification

The Merative MarketScan Commercial and Medicare Supplemental claims database (2008–2017) was queried for men ages >18 years with a diagnosis of testis mass who underwent radical orchiectomy. These patients were stratified according to implant status: orchiectomy with no implant, orchiectomy with CI, and orchiectomy with SI. For men with multiple implant records, we selected the earliest date following their orchiectomy.

We recorded demographic information at the time of orchiectomy including age, Charlson comorbidity index, geographic region, urban vs rural status, surgical setting (outpatient vs inpatient) and insurance type. These variables were chosen as baseline patient health, regional differences, and insurance coverage may all be confounding factors that affect access to surgery. We also recorded all healthcare records 30 days before orchiectomy (preoperative), at the time of orchiectomy (perioperative), and for 90 days following orchiectomy (postoperative). For patients who had an implant at a later date, we recorded all healthcare records in these same periods surrounding their implant procedure. If the orchiectomy postoperative period and the implant preoperative period overlapped (that is, if the implant took place sooner than 120 days following orchiectomy), then the orchiectomy postoperative period was truncated 30 days prior to the implant.

Patients were sub-stratified by whether they received a diagnosis of metastatic cancer and underwent additional cancer treatment (chemotherapy, radiation, retroperitoneal surgery) within one year of their orchiectomy. The rationale for this sub-stratification was that patients with metastatic cancer would likely require more imaging, laboratory studies to monitor their disease, thus confounding the cost analyses. We then looked across each patient’s entire follow up period to see who required an explant.

Exclusion criteria included patients without one year of enrollment data following orchiectomy (for those with CI) or implant (for those with SI). Patients with an implant record that predated their earliest orchiectomy record were also excluded as it was assumed they had an earlier orchiectomy not captured in the data.

Institutional Review Board approval was not obtained for this study given we utilized a publicly available insurance claims dataset.

### Statistical analysis

We ran multivariate linear regressions of all healthcare costs in the three time periods of interest (preoperative, perioperative, and postoperative) surrounding the radical orchiectomy and the implant if it was performed during a separate encounter.

In order to calculate preoperative cost, preoperative imaging and laboratory studies within 1 month leading up to and including the day of surgery were identified. Imaging studies included chest x-ray, chest, abdominal, or pelvic computerized tomography (CT) scan, abdominal, pelvic, or brain magnetic resonance imaging (MRI), and scrotal ultrasound (US). Laboratory studies included alpha fetoprotein (AFP), beta human chorionic gonadotropin (beta-hCG), lactate dehydrogenase (LDH), complete blood count (CBC), and basic or comprehensive metabolic panel (BMP or CMP).

In order to calculate perioperative cost, the Current Procedural Terminology (CPT) codes were obtained for radical orchiectomy and insertion of prosthesis, if applicable.

As part of the postoperative cost calculation, 90-day clinical outcomes were measured by rates of reoperation, readmission, emergency department (ED) presentation, and urology-related outpatient visits to broadly cover the different points of care the patients could interact with the health system in the postoperative setting. Costs also accounted for the aforementioned imaging and laboratory studies. Reoperation included the CPT codes for scrotal exploration, testicular prosthesis explantation, and testicular prosthesis re-implantation. For patients with metastatic disease, costs of adjunct treatments included the CPT codes for chemotherapy, radiation, and retroperitoneal lymph node dissection (RPLND).

To account for variations in length of each time period between patients, costs were standardized to person-months for the preoperative and postoperative periods. Because the perioperative period was only one day, no standardization was needed. Chi-squared and Kruskal-Wallis tests were utilized for categorical and continuous demographic variable comparisons, respectively. Multivariable linear, Poisson, and logistic regression models were employed for rate ratio comparisons of care utilization and costs. All models included age and the presence of cancer treatment as additional covariates. Full model outputs are available in S1 Table.

## Results

We identified a total of 8803 patients underwent orchiectomy. 8564 patients (97.3%) did not receive an implant, 190 patients received CI (2.1%), and 49 patients received SI (0.6%). Testicular prosthesis utilization rate was 2.7% (n = 239/8803).

Demographic characteristics of the total cohort and subgroups stratified by implant status are listed in Table 1. There was no difference in age (p = 0.398), Charlson Comorbidity Index (p = 0.495), insurance coverage (p = 0.135), surgical setting (p = 0.203), rate of metastasis (p = 1.000), or additional cancer treatment (p = 0.921) among patients who received CI versus SI. CI patients were significantly more likely to reside in an urban setting compared to SI (96% vs 86%, p = 0.013). Patients on the West Coast were more likely to receive CI (32%), whereas patients in the South were more likely to receive SI (47%), though this did not reach statistical significance (p = 0.089).

**Table 1:** Cohort demographics according to implant status.

Demographic	Total Cohort	No Implant	Concurrent Implant	Staged Implant	p-value
*Total*	8803 (100%)	8564 (97.29%)	190 (2.16%)	49 (0.56%)	-
*Age*	39.71 ± 13.9	39.87 ± 13.94	33.53 ± 10.42	35.2 ± 11.25	0.398
*Charlson Comorbidity Index*					
0	636 (7.22%)	621 (7.25%)	13 (6.84%)	2 (4.08%)	0.495
1	6325 (71.85%)	6134 (71.63%)	148 (77.89%)	43 (87.76%)	
2	1349 (15.32%)	1323 (15.45%)	23 (12.11%)	3 (6.12%)	
3+	493 (5.6%)	486 (5.67%)	6 (3.16%)	1 (2.04%)	
*Region*					
Northeast	1981 (22.5%)	1922 (22.44%)	49 (25.79%)	10 (20.41%)	0.089
Midwest	2078 (23.61%)	2044 (23.87%)	27 (14.21%)	7 (14.29%)	
South	2936 (33.35%)	2861 (33.41%)	52 (27.37%)	23 (46.94%)	
West	1629 (18.51%)	1560 (18.22%)	60 (31.58%)	9 (18.37%)	
Other	179 (2.03%)	177 (2.07%)	2 (1.05%)	0 (0%)	
*Population Density*					
Urban	7686 (87.31%)	7461 (87.12%)	183 (96.32%)	42 (85.71%)	**0.013**
Rural	1117 (12.69%)	1103 (12.88%)	7 (3.68%)	7 (14.29%)	
*Insurance Type*					
Comprehensive	293 (3.33%)	291 (3.4%)	2 (1.05%)	0 (0%)	0.135
EPO	155 (1.76%)	146 (1.7%)	9 (4.74%)	0 (0%)	
HMO	1009 (11.46%)	970 (11.33%)	31 (16.32%)	8 (16.33%)	
PPO	5371 (61.01%)	5227 (61.03%)	115 (60.53%)	29 (59.18%)	
POS	574 (6.52%)	563 (6.57%)	7 (3.68%)	4 (8.16%)	
POS with Capitation	60 (0.68%)	59 (0.69%)	1 (0.53%)	0 (0%)	
CDHP	471 (5.35%)	460 (5.37%)	11 (5.79%)	5 (10.2%)	
HDHP	339 (3.85%)	333 (3.89%)	3 (1.58%)	3 (6.12%)	
Missing	531 (6.03%)	515 (6.01%)	11 (5.79%)	5 (10.2%)	
*Surgical Setting*					
Outpatient	8519 (96.77%)	8283 (96.72%)	189 (99.47%)	47 (95.92%)	0.203
Inpatient	284 (3.23%)	281 (3.28%)	1 (0.53%)	2 (4.08%)	
*Cancer Status*					
Metastatic Disease	1150 (13.06%)	1125 (13.14%)	20 (10.53%)	5 (10.2%)	1.000
Additional Cancer Treatment	4319 (49.06%)	4201 (49.05%)	93 (48.95%)	25 (51.02%)	0.921

EPO: exclusive provider organization; HMO: health maintenance organization; PPO: preferred provider organization; POS: point of service, CDHP: consumer directed health plan; HDHP: high deductible health plan

Median preoperative, perioperative, and postoperative costs for the total cohort stratified by implant and cancer status are summarized in Table 2. Median perioperative orchiectomy cost for the no implant, CI, and SI groups were $5682 (3648–8554), $7823 (5403–10973), and $5380 (4130–10521), respectively (p<0.001). Median perioperative cost at the time of SI placement was $8180 (4920–14591) for a total perioperative cost of $13560 (5380 + 8180). Postoperative costs were eight times higher in patients who received additional cancer treatment for the no implant ($24264 vs $3710) and CI ($24686 vs $3210) cohorts.

**Table 2:** Median costs in each time period stratified by implant status and additional cancer treatment.

Time Period	Additional Cancer Treatment	No Implant	Concurrent Implant	Staged Implant (Orchiectomy Procedure)	Staged Implant (Implant Procedure)
*Preoperative*	No	n = 4363 $1262 (635–2623)	n = 97 $1144 (592–2514)	n = 24 $1280 (606–2469)	n = 24 $303 (83–1071)
	Yes	n = 4201 $1384 (737–2922)	n = 93 $1550 (732–3067)	n = 25 $1032 (503–2236)	n = 25 $509 (245–1889)
	Total	n = 8564 $1324 (681–2749)	n = 190 $1389 (654–2754)	n = 49 $1149 (545–2401)	n = 49 $432 (150–1694)
*Perioperative*	No	n = 4363 $5381 (3617–8692)	n = 97 $8157 (6038–11185)	n = 24 $5802 (4003–7954)	n = 24 $8083 (4817–13770)
	Yes	n = 4201 $5557 (3662–8429)	n = 93 $7130 (5288–10372)	n = 25 $5373 (4186–10953)	n = 25 $8180 (4932–20503)
	Total	n = 8564 $5682 (3648–8554)	n = 190 $7823 (5403–10973)	n = 49 $5380 (4130–10521)	n = 49 $8180 (4920–14591)
*Postoperative*	No	n = 4363 $3710 (1054–9932)	n = 97 $3210 (839–8287)	n = 24 $11051 (5375–21201)	n = 24 $837 (262–1788)
	Yes	n = 4201 $24264 (9143–56294)	n = 93 $24686 (6949–52679)	n = 25 $16451 (7448–27865)	n = 25 $1461 (718–3058)
	Total	n = 8564 $9405 (2276–32496)	n = 190 $7450 (2361–26897)	n = 49 $12421 (5547–21319)	n = 49 $1039 (483–2681)

90-day postoperative care utilization and costs are summarized in Table 3. CI patients were more likely to have a 90-day postoperative urology follow-up (59% vs 37%, p = 0.006) with an overall higher number of visits (268 vs 28, p = 0.030) compared to SI group during the implant period, but had similar follow-up rates (59% vs 73%, p = 0.065) compared to the SI patients during the initial orchiectomy period. Cost of readmission was also significantly higher in the CI group compared to the SI group during initially orchiectomy period ($25887 vs $8093, p = 0.019), but this was difference was negated if cost of median admission during the subsequent implant period ($13006) was considered.

**Table 3:** 90-day postoperative care utilization and costs.

*90-Day Care Utilization*	*Concurrent Implant*	*Staged Implant (Orchiectomy Procedure)*	*Rate Ratio (95% CI)*	*p-value*	*Staged Implant (Implant Procedure)*	*Rate Ratio (95% CI)*	*p-value*
*Emergency Department*							
Patients	51 (27%)	13 (27%)	0.984 (0.47–1.966)	0.965	7 (14%)	0.454 (0.177–1.966)	0.073
Visits	83	18	0.851 (0.495–1.381)	0.534	7	0.614 (0.258–1.381)	0.216
Median cost per visit (IQR)	$1032 (234–4403)	$949 (445–1940)	0.59 (0.175–1.988)	0.396	$1154 (412–2878)	1.126 (0.156–8.116)	0.907
*Urology*							
Patients	112 (59%)	36 (73%)	1.929 (0.981–3.993)	0.065	18 (37%)	0.404 (0.208–0.766)	**0.006**
Visits	268	111	1.289 (1.029–1.603)	**0.025**	28	0.650 (0.431–0.941)	**0.030**
Median cost per visit (IQR)	$126 (70–349)	$143 (68–884)	1.329 (0.83–2.129)	0.238	$90 (29–1059)	0.858 (0.37–1.988)	0.721
*Readmission*							
Patients	23 (12%)	4 (8%)	0.645 (0.182–1.783)	0.440	2 (4%)	0.309 (0.048–1.783)	0.120
Visits	40	8	1.15 (0.499–2.327)	0.718	2	0.575 (0.094–2.327)	0.445
Median cost per visit (IQR)	$25887 (13081–49511)	$8093 (7605–16219)	0.368 (0.164–0.827)	**0.019**	$13006 (12766–13246)	0.473 (0.104–2.153)	0.338
*Reoperation*							
Patients	6 (3%)	2 (4%)	1.305 (0.187–5.874)	0.749	4 (8%)	2.726 (0.673–5.874)	0.132
Visits	7	2	0.857 (0.128–3.546)	0.848	4	0.857 (0.224–3.546)	0.806
Median cost per visit (IQR)	$1361 (856–3102)	$5220 (3634–6807)	2.545 (0.302–21.44)	0.410	$1674 (865–8123)	1.486 (0.281–7.861)	0.360

Over the entire follow-up period, prosthesis explant rates were 4.7% for the CI and 14.3% for the SI cohort (p = 0.04) with a median time to explant of 166 (135–210) and 40 (9.5–141.5) days, respectively (p = 0.06; Table 4). Median cost of explant surgery was $2060 (IQR: 967–2880).

**Table 4:** Testicular prosthesis explantation rates.

	*Concurrent Implant*	*Staged Implant*	*p-value*
***Explantation Rate***	4.74% (n = 9/190)	14.29% (n = 7/49)	**0.039**
***Median time to explant (IQR)***	166 days (135–210)	40 days (9.5–141.5)	0.064

## Discussion

Testicular cancer is associated with high incidence and prolonged survival in young men. Subsequently, there are several efforts aimed towards evaluating and improving quality of life among this patient population. Testicular loss has been demonstrated to result in feelings of uneasiness, with one-third of men reporting that they miss their testicle and one-fourth of men reporting feelings of bodily shame [12]. One surgical option that may alleviate this identity crisis and restore self-image following the loss of a testicle is implantation of a testicular prosthesis [13]. It remains undetermined whether outcomes differ for patients who undergo concurrent versus staged prosthesis implantation. Herein we demonstrate that CI placement has less total perioperative cost, lower explant rate, and similar postoperative care utilization to SI.

Despite recommendation by the AUA guidelines and several studies demonstrating high patient satisfaction rates with testicular prostheses, many patients are not offered implantation [10, 11]. Specifically, the AUA guidelines state that patients should be counseled that they have the choice to elect for testicular prosthesis at the time of orchiectomy [2]. The rationale for this guideline is the high satisfaction rates coupled with low morbidity risk [10, 11]. Nevertheless, patients should be counseled on possible sequelae of infection, malposition, deflation, or need for explantation [14]. If patients decline concurrent prosthesis, they should be counseled that they can elect to undergo delayed insertion of testicular prosthesis later in their clinical course per the guidelines. Our results are in concordance with these findings, with a testicular prosthesis utilization rate under 3%. Men who have never been offered a prosthesis were significantly more likely to report feelings of loss (RR 2.0, 95% CI 1.3–3.0), and uneasiness or shame (RR 2.0, 95% CI: 1.3–3.2) compared to men who were offered a prosthesis but declined [12]. Thus, lack of discussion surrounding testicular prostheses with patients undergoing radical orchiectomy represents a significant missed opportunity.

One question that may arise during preoperative counseling is when to perform implantation. It has been previously demonstrated that concurrent insertion of testicular prosthesis during radical orchiectomy is safe, and this extra procedure does not increase the complication rate as determined by length of stay (p = 0.387), hospital readmission rates (p = 0.539), or reoperation (p = 0.999) compared to radical orchiectomy alone [15]. Another study demonstrated that concurrent implantation is safe even in the setting of adjuvant therapy, with no difference in complication rates between patients who did not undergo adjuvant treatment versus those who underwent chemotherapy (p = 0.75) or radiation (p = 0.83) [16]. Ours is the first study to directly compare the cost and clinical outcomes between a concurrent versus staged approach. We found that CI offers the benefits of lower perioperative cost by almost two-fold and lower explantation rates by almost three-fold. Reasons for testicular prosthesis explantation include infection, extrusion, rupture of prosthesis, or patient dissatisfaction [14]. Given the utilization of a claims dataset, reasons for explantation among our patients were not specified thus postulation regarding the difference between the two groups is limited.

The mean age of our cohort was 39 years, which is concordant with the known high incidence of testicular cancer in young men. Given that comorbidities increase with age, it was assumed that this young population would be less likely to have other medical conditions, and therefore we chose to specifically stratify our patients by presence of metastatic disease to account for confounding increased costs of additional cancer treatments. Specifically, 90-day postoperative costs were eightfold in patients who required additional cancer treatment in our cohort.

There were several limitations to this study. Firstly, prospective investigation is needed to confirm our findings as our study was retrospective in nature. Second, our data was obtained from an insurance claims dataset. Though a valuable resource, claims data are inherently limited due to their dependence on International Classifications of Diseases (ICD) coding, which may result in some variability and inaccuracy. More granular data is not available, and results generated from the dataset also only apply to the insured population.

## Conclusions

Preoperative counseling regarding testicular prostheses is an important component that urologists should discuss with patients undergoing radical orchiectomy for testicular cancer. CI placement has less total perioperative cost, lower explantation rates, and similar postoperative costs and utilization compared to SI.

## Supporting information

S1 Table Full model outputs from multivariable modeling. (DOCX)
