# Supplementary material for: Cost and utilization analysis of concurrent versus staged testicular prosthesis implantation for radical orchiectomy
Source: PLoS One. 2024 Jan 8;19(1):e0296735. doi: 10.1371/journal.pone.0296735 (PMC10773930; doi:10.1371/journal.pone.0296735)
Supplement: S1 Table — (DOCX) [file pone.0296735.s001.docx]

**Supplemental Table 1.** **Full model outputs from multivariable modeling.**

Effect of age represents increase of one year. Effect of group relative to non-implant patients, Effect of cancer treatment relative to people with no record of cancer treatment.

| Variable | Estimate | Std. Error | t value | Pr(>\|t\|) | Time |
| --- | --- | --- | --- | --- | --- |
| Intercept | 1348.629 | 270.749 | 4.981 | 0.000 | Pre |
| Age | 25.253 | 5.959 | 4.238 | 0.000 | Pre |
| Group (Later Implant (orch.)) | -543.676 | 1108.532 | -0.490 | 0.624 | Pre |
| Group (Later Implant (imp.) | -1259.061 | 1108.532 | -1.136 | 0.256 | Pre |
| Group (Sameday Implant) | -82.134 | 568.620 | -0.144 | 0.885 | Pre |
| CA Tx | 1235.718 | 165.076 | 7.486 | 0.000 | Pre |
| Intercept | 8422.136 | 472.946 | 17.808 | 0.000 | Peri |
| Age | -16.460 | 10.410 | -1.581 | 0.114 | Peri |
| Group (Later Implant (orch.)) | -689.670 | 1936.579 | -0.356 | 0.722 | Peri |
| Group (Later Implant (imp.) | 2036.804 | 1936.579 | 1.052 | 0.293 | Peri |
| Group (Sameday Implant) | 1373.309 | 993.363 | 1.382 | 0.167 | Peri |
| CA Tx | 649.562 | 288.366 | 2.253 | 0.024 | Peri |
| Intercept | 3599.159 | 1023.320 | 3.517 | 0.000 | Post |
| Age | 32.831 | 22.490 | 1.460 | 0.144 | Post |
| Group (Later Implant (orch.)) | -4123.498 | 4176.140 | -0.987 | 0.323 | Post |
| Group (Later Implant (imp.) | -10351.127 | 4090.309 | -2.531 | 0.011 | Post |
| Group (Sameday Implant) | 3868.935 | 2126.740 | 1.819 | 0.069 | Post |
| CA Tx | 13409.869 | 622.103 | 21.556 | 0.000 | Post |
